# Successful control of umbilical hidradenitis suppurativa with staged CO_2_ laser marsupialization

**DOI:** 10.1016/j.jdcr.2026.06.040

**Published:** 2026-06-25

**Authors:** Nicole Flores, Sydney R. Resnik, Barry I. Resnik

**Affiliations:** a Wayne State University School of Medicine, Detroit, Michigan; b Dr. Phillip Frost Department of Dermatology and Cutaneous Surgery, University of Miami Miller School of Medicine, Miami, Florida; c Resnik Skin Institute, Aventura, Florida

**Keywords:** atypical HS presentation, biologic therapy, CO_2_ laser marsupialization, hidradenitis suppurativa (HS), umbilical HS

## Introduction

Hidradenitis suppurativa (HS) is a chronic, relapsing inflammatory disease of the follicular unit, characterized by painful nodules, abscesses, and sinus tracts. It most frequently involves intertriginous areas such as the axillae, groin, and perianal region, with a reported prevalence of 1% to 4% in the general population. Known risk factors include obesity, smoking, and genetic predisposition. Although HS has a relatively predictable anatomic distribution, atypical presentations can occur and may delay diagnosis. Umbilical involvement is rarely reported in the literature. Recognizing uncommon sites is essential for accurate disease staging and appropriate management. We present a case of Hurley stage III HS with umbilical involvement managed successfully using combined surgical and biologic therapy.

## Case presentation

A 52-year-old Hispanic man with Hurley stage III HS presented with chronic lesions affecting the right axilla, bilateral inguinal folds, scrotum, waistline, and the umbilicus. He had a history of obesity treated with bariatric surgery. Diagnosed in 2015, he had previously been treated with oral prednisone, dapsone, isotretinoin, and lifestyle interventions. Isotretinoin provided temporary relief, but symptoms recurred. He had not previously used biologic therapy.

On examination, he exhibited extensive scarred plaques with tunnels involving the inner thighs, scrotum and perineal skin, and the umbilicus. The umbilical lesion was a tender, fluctuant, erythematous plaque without active drainage. Due to the atypical location, an ultrasound was obtained to exclude intra-abdominal extension, which showed no deep tissue involvement.

He was counseled on smoking cessation, a low-dairy/low-carbohydrate diet, antiseptic body washes, and zinc supplementation. Oral clindamycin and rifampin were initiated and then discontinued after initiation of adalimumab.

Over the next several months, adalimumab helped bring his disease under better control. He underwent Staged CO_2_ Laser Marsupialization (SCLM) of lesions on the left scrotum, perineum, and bilateral inguinal folds with excellent result. However, the umbilical lesion continued to wax and wane.

**Fig 1:**
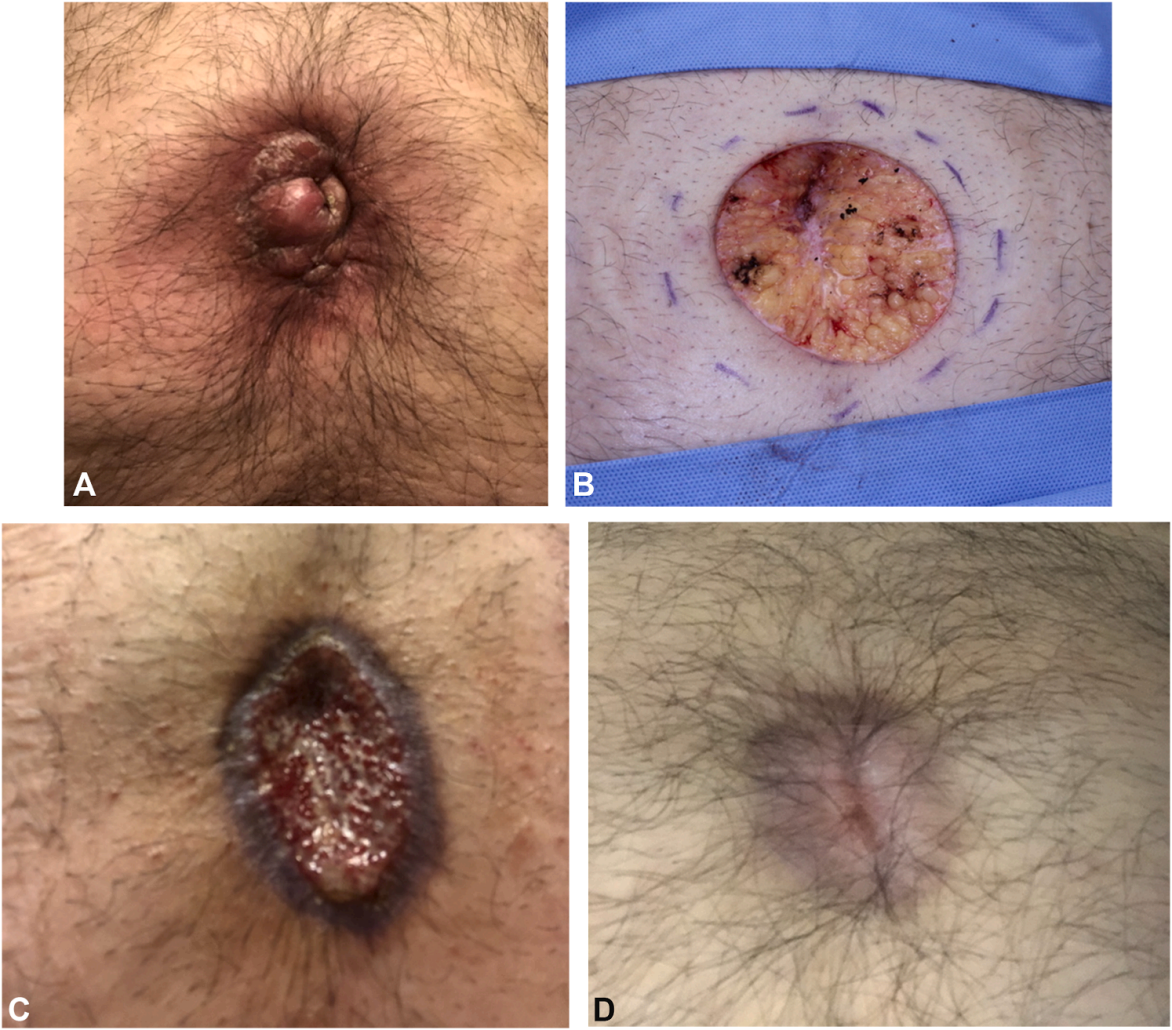
Hidradenitis suppurativa of umbilicus treated with SCLM. **A,** Preoperative; **B,** Immediately post-operative; **C,** Healing process; **D,** Fully healed.

In 2018, he reported a flare of the umbilical lesion, characterized by recurrent tenderness, erythema, and purulent drainage. Incision and drainage followed by silver nitrate application was performed in-office, providing only temporary relief. In 2019, he underwent definitive SCLM of the umbilicus (Fig 1, *A*-*D*) as well as the right axilla.

The procedure was performed using a Sharplan 1030 CO_2_ laser (Lumenis) set at 30 W in continuous-wave mode. The laser was used to excise the affected tissue to healthy margins. Although an electrocautery unit was available for hemostasis in the event of arteriolar bleeding, it was not required, and coagulation was achieved with the laser alone.

Postoperatively, the wound was dressed with a nonadherent petroleum jelly–coated pad secured with gauze and tape. During the first 12-24 hours, a more absorbent dressing was used to accommodate expected exudate from the tumescent anesthetic solution. Thereafter, the patient performed once-daily dressing changes using the smallest occlusive dressing necessary as the wound progressively contracted and healed. Wound healing was uncomplicated and proceeded by secondary intention without infection or delayed healing. The patient did not report moderate or severe postoperative pain, and discomfort was well tolerated with routine postoperative care.

Following surgery, the patient remained on adalimumab and adhered to lifestyle modifications. He experienced no new HS lesions in any of the surgical sites during follow-up through 2021. He was able to resume normal physical activity, including sports, without limitations.

## Discussion

While HS most commonly affects intertriginous regions, atypical presentations such as umbilical involvement may go unrecognized and delay diagnosis. In our case, the umbilical lesion was initially misinterpreted as a primary abscess by previous treating physicians. Given the anatomical location, differential diagnosis included urachal remnant infection, omphalitis, or extra sacrococcygeal pilonidal sinus (ESPS). Imaging was essential in ruling out intra-abdominal extension prior to surgical treatment.

Although umbilical HS is rarely reported, under-recognition may be more likely than rarity. Andersen et al reported that 7% to 10% of patients with HS had umbilical symptoms such as erythema, pruritus, or discharge, underscoring the importance of high clinical suspicion in chronic umbilical lesions.^1^ Other reports have documented HS presentations on the face^2^ and cervicofacial region,^3^ reinforcing that the disease may occur outside traditional intertriginous zones.

Surgical management remains a cornerstone in Hurley stage II-III HS, especially when tunnels and fibrosis are present. Traditional wide en bloc excision offers definitive removal but is associated with prolonged wound healing, increased morbidity, and high recurrence rate.^4^^,^^5^ SCLM provides a tissue-sparing alternative with a low recurrence rate. Hazen and Hazen reported favorable outcomes with this approach, including reduced recurrence and improved functional recovery in moderate-to-severe HS.^6^

In the present case, the procedure qualified as SCLM because the diseased tissue was sequentially vaporized and marsupialized along the course of the interconnected sinus tracts rather than removed as a single block of tissue. Using the CO_2_ laser allowed progressive removal of the involved tissue with direct visualization of the underlying tunnels until healthy tissue margins were reached. This approach permits identification and treatment of multiple branching tracts during the procedure while preserving adjacent uninvolved structures. Although the final postoperative appearance demonstrated complete removal of the affected tissue, the technique itself involved stepwise identification and destruction of the individual sinus tracts and epithelialized tunnel walls, which is the defining feature of staged laser marsupialization rather than simple excision.

Deroofing was considered but was less suitable in this case because the lesion contained multiple interconnected sinus tracts within the confined umbilical tissue, which would have required extensive unroofing and potentially incomplete removal of epithelialized tunnel structures. In addition, the patient elected to proceed with SCLM after discussion of treatment options. Compared with scalpel excision, the CO_2_ laser provides superior precision and hemostasis, enabling targeted removal of diseased tissue while minimizing collateral damage to surrounding structures. This precision is particularly advantageous in anatomically sensitive areas such as the umbilicus, where preservation of contour and reduction of postoperative morbidity are important considerations. Resnik and Hazen have similarly emphasized the advantages of CO_2_ laser-based approaches in cosmetically or functionally sensitive regions.^7^

In our patient, this technique provided effective and durable disease control, including at the umbilicus, where wide excision could have resulted in greater tissue loss and more challenging wound healing. Biologic therapy with tumor necrosis factor-alpha inhibitors, such as adalimumab, also plays a central role in the management of moderate-to-severe HS by targeting the underlying inflammatory pathways. However, chronic lesions containing fibrotic sinus tracts are frequently refractory to medical therapy alone. A combined surgical and biologic approach, as employed in this case, can optimize outcomes and prolong remission.

Finally, lifestyle factors, including obesity and smoking, are well-established contributors to HS severity and recurrence. Comprehensive patient education and ongoing lifestyle modifications were essential components of this patient’s long-term disease control.

This case highlights the successful treatment of severe, multiregional HS with rare umbilical involvement using a multimodal approach: SCLM, adalimumab, and lifestyle modifications. It emphasizes the importance of recognizing atypical HS sites, utilizing imaging for pre-surgical planning, and considering combined medical-surgical strategies in refractory disease. Increased awareness of atypical HS presentations is crucial for timely diagnosis and effective treatment.

## Conflicts of interest

None disclosed.
